# Informing environmental health and risk priorities through local outreach and extension

**DOI:** 10.1007/s10669-022-09864-0

**Published:** 2022-06-02

**Authors:** Khara Grieger, Christopher L. Cummings

**Affiliations:** 1 Department of Applied Ecology, NC State Extension, North Carolina State University, Campus Box 7617, Raleigh, NC 27695 USA; 2 Genetic Engineering and Society Center, North Carolina State University, Campus Box 7565, Raleigh, NC 27695 USA; 3 Gene Edited Food Program, Iowa State University, 510 Farm House Lane, Ames, IA 50011-1054 USA; 4 US Army Engineer Research and Development Center, Concord, MA USA

**Keywords:** Environmental health, Risks, Priorities, North Carolina

## Abstract

Our society is currently facing an unprecedented number of environmental and societal challenges. Stakeholder and community engagement can help identify priority issues and needs at local levels. One approach to engage stakeholders and communities in the contexts of environmental, health, and societal challenges is to leverage outreach and extension programs. Within this context, and to help identify priority issues to focus subsequent research and extension programs in North Carolina (NC), a survey was conducted with extension agents to identify priority issues as they relate to environmental health and risks and related needs. Based on responses from 66 study participants that represented half of the 100 NC counties, we found that *Water pollution*, *Flooding*, *Natural resources management*, and *Engaging stakeholders* were top priority issues across all environmental health and risk topics. Participants also identified that practices of *Engaging stakeholders* as well as *Assessing*, *Managing*, and *Communicating risks* were increasingly important. Participants indicated they needed a moderate-to-significant amount of guidance across a range of areas related to assessing, managing, communicating, and making decisions regarding environmental health and risk topics, as well as engaging with local communities. Outcomes from this work can not only help inform subsequent research and outreach efforts at local scales, but this work demonstrates a simple, low-cost approach to elicit perspectives and priorities can be leveraged in other states and regions with established stakeholder and community outreach programs more broadly.

**Supplementary Information:**

The online version contains supplementary material available at 10.1007/s10669-022-09864-0.

## Introduction

Our society is currently facing an unprecedented number of environmental and societal challenges, including climate change, loss of biodiversity, environmental pollution, deforestation, and depletion of many critical ecosystem services, among others (Landrigan and Fuller 2015, Burke et al. 2018, Hall-Spencer et al. 2019, Chase et al. 2020). Many of these challenges may be considered “wicked problems,” given their complex, interdependent relationships among several environmental and social systems, there may be no single cause (and therefore no single ‘silver bullet’ solution), and therefore may require collaboration across a diverse set of actors (Ranabahu 2020; Lawrence et al. 2022). In these instances, it is becoming increasingly clear that interdisciplinary efforts are urgently needed to address our wicked problems, including research and engagement efforts that cross boundaries between technical and natural sciences, engineering, as well as social sciences and humanities (Kuzma et al. 2020 ; Kuiken et al. 2021). We now have several case studies that clearly demonstrate that developing science and new technological solutions are not enough to tackle our complex environmental and societal challenges (Kuzma 2018; Grieger et al. 2019; Cao et al. 2020, Kuzma et al. 2020). Rather, we also need to couple scientific, research, and innovation efforts with stakeholder engagement and communication while also building trust among societal actors (Grieger et al. 2012, 2021; O’Brien et al. 2013; Kuzma and Grieger 2020). Moreover, addressing environmental challenges at all levels—from local to regional to global scales—often require robust communication, collaboration, and coordination between a range of stakeholder groups, such as scientific experts, local/regional government officials, land use managers, advocacy groups, and community members (IRGC 2017; Renn 2015; IRGC 2018).

Engaging stakeholders within broader efforts to identify and mitigate environmental, health, and societal risks helps ensure that research and policy efforts align with societal needs and wants. In addition, engaging stakeholders within risk identification and governance will also help ensure that a broader and perhaps more representative set of perspectives are included in such efforts (Dendler and Böl 2021). At the same time, stakeholder engagement activities need to be tailored and customized for individual case studies while considering surrounding socio-economic and political contexts, complexities and nature of the case study, as well as diverse set of stakeholders and community members that may be impacted or involved (Renn 2015). For example, consultation-based engagement activities may be used in some circumstances to better understand views of stakeholders or community members to inform policy decisions (e.g., views of the severity of chemical contaminants in a local water body, views of alternative remediation techniques to clean up chemical contaminants). In other instances, stakeholders and community members may need to be engaged more intensely through deliberation activities, to co-develop knowledge or solutions to a given issue. Overall, the type and nature of engagement activities will depend on the goals of engagement and the stakeholders (and associated communities) that are impacted.

One approach to engage stakeholders and community members within contexts of environmental, health, and societal challenges is to leverage and work with cooperative extension programs that have a mission of translating knowledge and research from academic settings to non-academic stakeholder groups at local scales. In the United States (US), there are more than 100 land-grant colleges and universities that incorporate extension components into their institutional structures in addition to traditional concentrations of academic teaching and research (National Institute of Food and Agriculture (NIFA) 2022). According to the National Institute of Food and Agriculture, “extension provides non-formal education and learning activities to people throughout the country—to farmers and other residents of rural communities as well as to people living in urban areas. It emphasizes taking knowledge gained through research and education and bringing it directly to the people to create positive changes” (National Institute of Food and Agriculture (NIFA) 2022). Among other aspects, university-based extension is tasked with identifying emerging challenges and research questions and to develop solutions that help improve our society, and extension agents are employees of extension programs who help connect academic research with local communities to address various issues. For these reasons, university-based extension programs may be ideal for readily connecting with a range of stakeholders and local community members to identify legacy, new, or emerging environmental, health, and/or societal risks, given their established networks and already-existing mission of translating research knowledge to non-academic audiences. We argue that improved use of bottom-up social scientific surveying of local community stakeholder can enable extension programs and networks to better fulfill their shared mission to improve risk outlooks and may also help to identify priority issues and needs that may otherwise go unnoticed by leadership. Such surveying therefore can serve as a vital resource for collecting regional risk research prioritization data to better inform policymaking and funding decisions through robust and directed social scientific investigation.

With this background, we conducted a survey to identify priority issues and needs as they related to environmental health and risk challenges in the state of North Carolina (NC), drawing from perceptions and perspectives of extension agents across the state. In many ways, NC serves as a representative testbed for risk issues in other regions of the country. This is because NC has faced a wide range of environmental health and risk issues in recent years, including impacts of several severe and costly hurricanes that led to widespread flooding, industrial chemical pollution, and on-going issues with nutrient-run-off and eutrophication (Center for Human Health and the Environment 2022; Environment North Carolina 2022, EPA 2022), similar to other states and regions. NC is also racially and ethnically diverse and boasts a wide variety of geographic regions including a major mountain range, deciduous forest, agricultural plains, and the second largest estuarine system and coastline of the United States. Further, NC currently has one of the largest extension programs in the country, with offices and agents positioned in each of the state’s 100 counties, and therefore is well-positioned to identify top environmental health and risk issues and needs across the state. While there have been numerous studies that have identified environmental health and risk issues on global and regional scales (Bernhard et al. 2013; Woods et al. 2016; Rocks et al. 2017; Wu et al. 2017; Morris et al. 2020; Wolfson et al. 2020; Fu et al. 2021; Wang et al. 2021), there have not yet been any published studies that have focused on views of stakeholders located with NC specifically to the best of the authors’ knowledge.

For these reasons, we developed and disseminated a survey in the spring of 2021 among extension agents to identify priority issues and needs as they relate to environmental health and risk issues in NC. Our work was guided by the overarching research question, “What are the priority environmental health and risk issues reported by extension agents across North Carolina?” Outcomes from this work can not only help inform subsequent research and outreach efforts on local scales, but this work demonstrates a simple, low-cost, bottom-up approach to elicit perspectives and priorities on risks which is easily transferable to other states and regions with established stakeholder and community outreach programs.

## Methods

### Survey development

The survey was developed using Qualtrics, which is an online survey platform that allows for customizable questions, survey progress tracking, mobile formatting, and results downloads. The survey was cross-sectional in nature and was conducted anonymously with no identifying information collected and consisted of multiple-choice and open-ended questions that asked participants to report their perceptions and views on a range of environmental health and risk topics (Cummings 2017a). Survey questions were grouped into four broad categories: (1) Understanding Priorities; (2) Guidance and Assistance; (3) Information and Collaborators; and (4) Respondent Information. To better assist others who may conduct similar inquiry in the future, we provide an overview of the survey questions below as well as the complete list of survey questions in the Supplementary Information (SI).

#### Understanding priorities

Study participants first responded to survey questions that investigated their views of how important different categories of environmental health and risk topics are in relation to one another given the community(ies) they serve. The list of topics and sub-topics included in the survey were based on environmental health and risk issues that emerged from the broader literature on environmental health, risk, and sustainability, including several studies that identified priorities in environmental health and risk. Further, we also leveraged the author’s own experiences and connections with extension and community groups in NC focused on environmental risk issues. Participants were first asked to respond to the following question: “To begin, we would like to know your views of how important each of the following Soil, Water, Air Pollution, and Contamination issues are right now to your community(ies) you serve” and were referred to a list of topics and sub-topics (shown in Table 1). The same question was then posed to participants for the topics of *Ecosystems & Land Use Management*, *Emerging Societal Issues*, and *Cross-Cutting Issues* (see Table 1 for associated sub-topics). For each of these topics and sub-topics, participants rated the level of importance using a 7-point semantic differential scale (1 = Not at all important, 7 = Extremely important). Study participants were also invited to provide additional environmental health and risk-related topics they thought were important and were able to elaborate on their responses in open-ended questions.

**Table 1:** List of environmental health and risk-related topics and sub-topics that study participants rated according to importance, where participants rated the areas according to degree of importance using a 7-point scale (1 = Not at all important, 7 = Extremely important)

Environmental health and risk topic	Sub-topic
Soil, Water, Air Pollution, and Contamination	Air pollution
	Soil pollution
	Water pollution (e.g., contamination of drinking water and natural waters)
	Pollution from industrial chemicals (e.g., Perfluoroalkyl and Polyfluoroalkyl Substances, PFAS)
	Pesticides and pesticide management (e.g., glyphosate)
	Pollution from municipal solid waste (e.g., plastics in the environment
	Other topics related to soil, water, and air pollution (please specify)
Ecosystems & Land Use Management	Climate change
	Flooding, sea level rise, and/or coastal erosion
	Biodiversity loss
	Deforestation
	Fisheries management
	Natural resources management
	Sustainable agriculture and food security
	Other topic related to ecosystems and land use management (please specify)
Emerging Societal Issues	Micro-plastics and nano-plastics (e.g., in the environment and drinking water sources)
	Coal ash and managing coal ash spills
	Genetically-modified or genetically-engineered (GE) organisms (e.g., GE crops or gene drive for conservation)
	Nanotechnology and/or nanomaterials (e.g., in food and agriculture products)
	Solid waste management (e.g., plastics recycling, composting)
	Renewable energy
	Other topics related to emerging issues in society (please specify)
Cross-Cutting Issues	Assessing risks
	Managing risks
	Communicating risks
	Making decisions about risks
	Engaging stakeholders
	Other cross-cutting issue (please specify)

Next, participants were asked to indicate which environmental health and risk-related topic is of greatest priority, by responding to the question “Of all the environmental health and risk issues listed above, which one is the biggest issue you feel your community(ies) currently faces.” Similarly, participants were asked to indicate which topic they would expect to be the most important in the next 5 years, by responding to the question “Of all environmental health and risk issues listed above, which one is the biggest issue you expect to face in the next 5 years in your community(ies)?” See Section A of the SI for a complete list of all survey questions in this section.

#### Guidance and assistance

Next, study participants responded to survey questions that aimed to identify areas in which they needed guidance and/or assistance to respond to environmental health and risk-related issues.^1^ In this section, study participants were first asked, “Of all environmental health and risk issues listed above, which one is the biggest issue you need guidance or assistance to mitigate in your community(ies)?” referring to the list provided in Table 1. Participants were able to elaborate on their responses in an open-ended question. Next, participants rated the degree they would like guidance in a variety of areas, by responding to the question “To what degree would you like guidance in the following areas?” and were referred to list shown in Table 2. Participants then rated these items according to the degree they needed guidance, using a 7-point semantic differential scale (1 = Very little guidance needed, 7 = A great deal of guidance needed, 0 = No guidance needed). Participants were able to report additional areas where they needed guidance in an open-comment field.

**Table 2:** List of areas that study participants may need guidance. Participants rated the areas according to degree of guidance needed using a 7-point scale (1 = Very little guidance needed, 7 = A great deal of guidance needed, 0 = No guidance needed)

Areas that participants may need guidance or assistance
Developing Extension-related guidance materials
Developing Extension-related communication and outreach materials
Communicating with community members
Engaging with community members
Identifying high-risk populations
Identifying topics of concern within local communities
Identifying best contact personnel for additional guidance and/or advice
Understanding and evaluating environmental health and risks
Managing environmental health and risk topics
Communicating environmental health and risk topics
Making decisions for dealing with environmental health and risk topics
Other (please specify)

Next, Participants were asked to indicate if they would be interested in attending a number of professional development events in their field, including Field days, In-person workshops, Web-based workshops, In-person focus groups, Web-based focus groups, Phone/virtual meetings, Certification programs (virtual or in-person), One-on-one or small group training (virtual or in-person), and Other (please specify). For each of these events, participants provided responses of “Yes,” “No,” “Maybe,” or “I don’t know” responses to express interest in attending.

#### Information and collaborators

Study participants were also asked to indicate the information sources they use to identify new and/or emerging environmental health and risk issues as well as current and future collaborators to mitigating these issues.^2^ In this section, study participants were first asked to respond to the following question: “How often do you use each of the following information sources when identifying a new or emerging environmental health and risk issue?” and were referred to the information sources shown in Table 3. Similar to previous questions, participants rated each of the sources using a 7-point semantic differential scale (1 = Rarely use, 7 = Very frequently use, 0 = Do not use) and they had the option to report additional information sources they use in an open-comment question.

**Table 3:** List of information sources that participants may use to identify environmental health and risk issues, where they indicated their frequency of use on a 7-point scale (1 = Rarely use, 7 = Very frequently use, 0 = Do not use)

List of information sources to identify environmental health and risk issues
Academic research (e.g., scientific articles)
Internal research (e.g., your own research or extension-related activities)
Extension publications
Publicly available data and information
Professional or extension networks
Stakeholder or community feedback
Feedback from other extension agents or specialists
Social media
Mainstream media coverage
Personal experience
Other (please specify)

Next, study participants were asked which stakeholder and community groups they currently work with, and which groups would they consider to work with in the future (either formally or informally), by responding to the question “To identify, communicate, and/or respond to environmental risks, which groups are you currently working with, and which groups would you consider working with in the future, either formally or informally?” These groups included the following: Local community groups, Individual members of the public, Local government, State government, Federal government, Trade unions, Private businesses, NGOs, Indigenous populations, Academic and research institutions, and Other (please specify). For each of these groups, participants could indicate if they currently work with or would consider working with these groups using Yes/No responses (scored as 1 = No, 2 = Yes).

#### Respondent information

At the end of the survey, study participants were asked to indicate the county(ies) they currently serve using a drop-down menu, and their area of expertise using a multi-choice question with options of Agriculture & Food, Community, Forestry Resources, Health & Nutrition, Home & Family, Lawn & Garden, Soil, Water & Air, and Other (specify) to align with established extension program areas. Participants were also asked to indicate their preferences for receiving information from colleagues, using a multiple-choice question with options of Email, Phone, Virtual meetings, Print mailers, Web-based materials, and Other (please specify). Finally, participants were able provide any final comments and thoughts relevant for environmental health and risk topics in the county(ies) they serve, using an open-ended question.^3^

### Participant identification and outreach

As this study sought to characterize priority areas and needs according to extension agents in North Carolina, participants were identified through North Carolina State Cooperative Extension Service’s online directory (https://www.ces.ncsu.edu/directory/). Individuals who were working with or associated with extension program in areas of Agriculture & Food, Forest Resources, Health & Nutrition, Home & Family, Lawn & Garden, and Soil, Water & Air were identified, and candidate names and contact information were compiled. After searching the extension directory, a list of 327 potential survey participants that represented a range of extension areas related to environmental health and risk issues across all counties were identified. After obtaining IRB approval through NC State (IRB protocol 23998), potential study participants were contacted via email and invited to participate in the study. Reminder emails were sent approximately two weeks after initial emails were sent. The outreach email included an overview of what the survey entailed (i.e., confidential and anonymous survey, questions that were multiple-choice and open-ended, using Qualtrics), as well as potential benefits of participating and information on how the results were handled.

### Survey dissemination and data collection

All study candidates were able to directly access the survey using a link included in the outreach email. The survey was distributed to study participants in the end of April 2021, and the survey remained open until mid-May 2021. Study participants were required to provide consent to participate in the study prior to starting the survey. Out of the 327 potential study participants that were identified and contacted to partake in the survey, 87 participants provided consent and completed part of the survey. In total, 66 participants completed the entire survey, and therefore the total number of participants considered to complete the survey was 66. It should be mentioned here that while 66 participants completed the entire survey, only 52 respondents completed every single item throughout the survey. Out of the 66 participants who completed the survey, the distribution of these participants according to areas of expertise are as follows: Agriculture & Food (*n* = 27, 53%), Lawn & Garden (*n* = 7, 14%), Community (*n* = 6, 12%), Other (*n* = 4, 8%), Forestry Resources (*n* = 2, 4%), Health & Nutrition (*n* = 2, 4%), Soil, Water, & Air (*n* = 2, 4%), and Home & Family (*n* = 1, 2%) (see Table A14 in SI). Of the participants who selected the expertise area of Other, they further specified these areas as 4-H Youth Development, Therapeutic Horticulture, Ecosystem protection (including watersheds and water quality protection), Natural Resource and Conservation, Field Crops, Horticulture, Community and School Garden/Local Food/Farm to School, and Environmental Assessment (Table A14 in SI). In addition, these participants represented half of all counties (*n* = 50) in North Carolina (Table A13 in SI).

After the study period ended, the Qualtrics survey was closed so that participants were no longer able to access the online survey. Following the study, all participants were sent an email to thank them for their participation and were provided details in terms of how the results of the survey would be used in future research, extension, and outreach work related to environmental health and risk.

### Survey analysis

After the completion of the study, responses were exported for analysis in SPSS version 26. For the multiple-choice questions, univariate descriptive statistics were calculated, including mean and standard deviation, of responses from the 66 participants who completed the survey. The mean scores and standard deviation values were then plotted to better visualize distinctions across measures. For the open-ended response questions, participants’ responses were exported and treated qualitatively for descriptive purposes.

## Results

### Importance of environmental health and risk issues

**Fig. 1:**
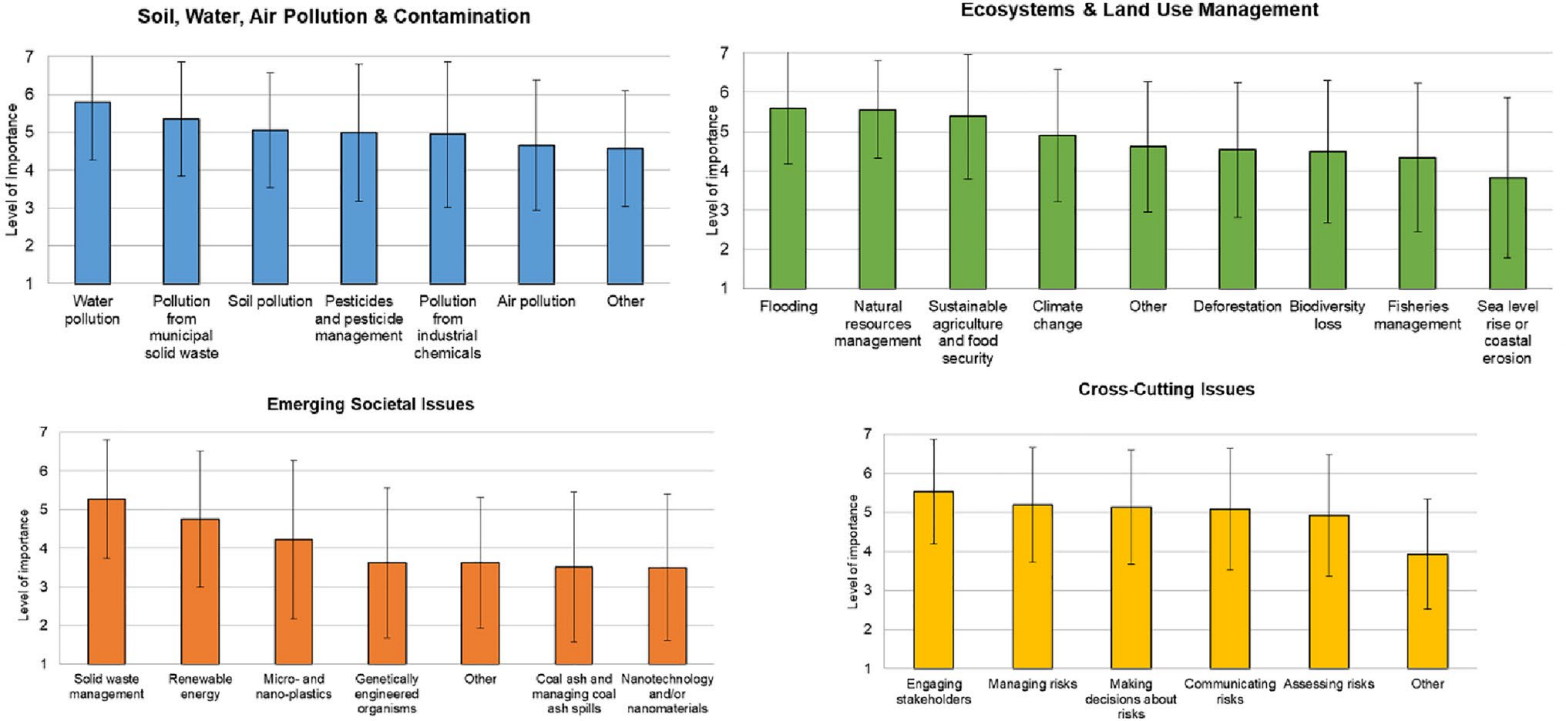
Importance ratings related to environmental health and risk issues faced by study participants and the communities they serve. Responses to “To begin, we would like to know your views of how important each of the following issues are right now to your community(ies) you serve,” (1 = Not at all important, 7 = Extremely important)

When asked to rate the importance of a range of environmental health and risk issues currently faced by communities served by study participants, most issues were rated between neutral and very important (Fig. 1). No environmental health and risk issue was rated less than neutrally important. Over the four topic areas of environmental health and risks, participants rated issues within *Soil, Water, Air Pollution & Contamination* as slightly more important than the other topic areas (*Ecosystems & Land Use Management*, *Emerging Societal Issues*, and *Cross-Cutting Issues*) (Fig. 1, see also Tables A1–A4 in SI). In fact, *Water pollution* was rated the highest across all risk issues between moderately and very important (Mean = 5.79, Standard deviation 1.54) (Table A1 in SI). In addition, *Flooding* (*M* = 5.6, SD = 1.42), *Natural resources management* (*M* = 5.56, SD = 1.24), and *Engaging stakeholders* (*M* = 5.53, SD = 1.34) (Fig. 1, Tables A2, A4) were rated as the next most important issues to study participants. Lowest priority issues rated by participants were *Sea level rise* (*M* = 3.82, SD = 2.05), *Nanotechnology and/or nanomaterials* (*M* = 3.5, SD = 1.90), and *Other cross-cutting issues* (*M* = 3.93, SD = 1.41), with participants specifying *Other* pertained to (i) distinguish between perceived and actual risks, (ii) not repeating mistakes with glyphosate, (iii) defining risks, and (iv) identifying issues that require stakeholder engagement.

Within the *Soil, Water, Air Pollution & Contamination* category, study participants rated *Water pollution* as very important, followed by *Pollution from municipal solid waste*, *Soil pollution*, *Pesticides and pesticide management*, and *Pollution from industrial chemicals* which were rated as moderately important (*M* = 4.94–5.35, SD = 1.52- 1.92) (Fig. 1, Table A1 in SI). As one participant mentioned in a subsequent question to further elaborate on a priority issue: “Right now PFAS seem to be the biggest concern in part of the county.” *Air pollution* and *Other* issues were rated as neutrally important (*M* = 4.57–4.65, SD = 1.54–1.72). Participants indicated that *Other* referred to a range of issues, including microplastics from biodegradable mulch, pollution from septic systems in coastal development, stormwater runoff, non-native species, trash and lack of recycling, animal manure and associated impacts from hurricanes, waste from wastewater treatment plants, and coal ash (Table A1).

Within the *Ecosystems & Land Use Management* category, study participants rated *Flooding*, *Natural resource management*, and *Sustainable agriculture & food security* between moderately and very important (*M* = 5.38–5.6, SD = 1.24–1.59) (Fig. 1, Table A2 in SI). This was followed by *Climate change*, *Other*, *Deforestation*, *Biodiversity loss*, and *Fisheries management*, which were rated as neutrally to moderately important (*M* = 4.34–4.9, SD = 1.66–1.89). *Sea level rise or coastal erosion* was rated as slightly to neutrally important (*M* = 3.82, SD = 2.05). In this category, participants indicated that *Other* referred a diverse set of issues including habitat protection, development within headwater regions, equitable food systems, loss of productive land to development, energy use, stabilizing shorelines, trash and little, timber harvest, protection of open spaces, weeds and wildlife competing with agricultural crops, and urban stormwater management (Table A2).

Within the *Emerging Societal Issues* category, study participants rated S*olid waste management* as moderately important (*M* = 5.27, SD = 1.54), followed by *Renewable energy*, *Micro- and nano-plastics* as neutrally to moderately important (*M* = 4.22–4.76, SD = 1.77–2.05) (Fig. 1, Table A3 in SI). *Genetically engineered organisms*, *Other*, *Coal ash and managing coal ash spills*, and *Nanotechnology and/or nanomaterials* were all rated as slightly to neutrally important (*M* = 3.5–3.62, SD = 1.70–1.94). Participants indicated that *Other* pertained to product lifecycles to minimize solid waste quantity and toxicity, carbon sequestration, and lack of public trust in scientific information (Table A3). Within the *Cross-Cutting Issues* category, study participants rated *Engaging stakeholders* as moderately to very important. Next, participants rated *Managing risks*, *Making decisions about risks*, *Communicating risks*, and *Assessing risks* as moderately important (*M* = 4.92–5.19, SD = 1.46–1.55) (Fig. 1, Table A4). *Other* issues in this category were rated as slightly important to neutral (*M* = 3.93, SD = 1.41), and as mentioned previously, pertained to (i) distinguish between perceived and actual risks, (ii) not repeating mistakes with glyphosate, (iii) defining risks, and (iv) identifying issues that require stakeholder engagement (Table A4).

Next, participants were given the option to list and describe any additional environmental health and risk-related topics that they think is important and not included in the previous list of issues. Six participants responded to this question and they mentioned topics related to land use management, wildfires, invasive plants, impacts of timber harvest, as well as environmental education for the greater public (Table A5 in SI). In addition, participants were given the option to elaborate on any of the environmental health and risk issues they considered to be highly important. Six participants responded to this open-ended question, and included the following topics as highly important: (i) loss of farm and forest land, (ii) recovering from hurricanes and impacts of climate change, (iii) PFAS concerns, (iv) protection of groundwater for individual and community wells, (v) trash and lack of recycling in rural areas, (vi) food deserts and food security (Table A5). For example, one participant indicated, “Some people in my county have not recovered from the last hurricane. Because of climate change, we are expected to get more and longer staying hurricanes.” Another study participant indicated, “I noted a high importance regarding environmental impacts on trash/waste. Living in rural NC, there is seemingly little to no emphasis on the benefits of recycling and reducing waste.”

### Current and future priorities

**Fig. 2:**
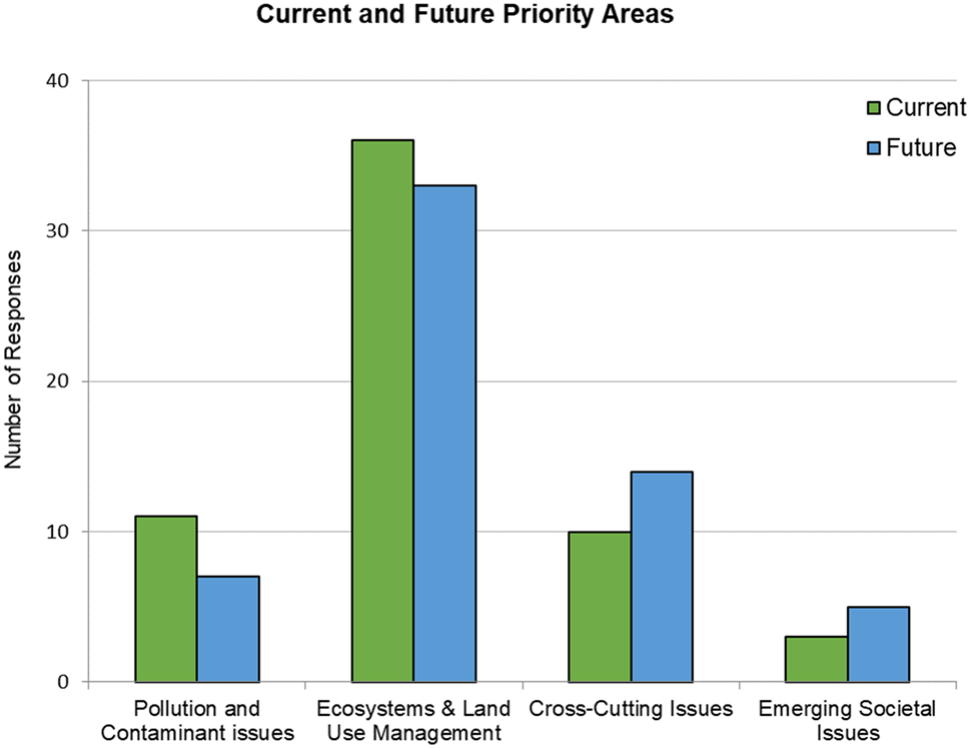
Current and future priority areas as identified by study participants. Responses to “Of all environmental health and risk categories listed below, which one is the biggest issue you currently face in your community(ies)?” and “Of all environmental health and risk categories listed below, which one is the biggest issue you expect to face in the next 5 years in your community(ies)?”

More than half of participants indicated *Ecosystems & Land Use Management* was the single biggest issue they currently face today (*n* = 36, 60% of participants) and expect to face in the future (*n* = 33, 55% of participants) (Fig. 2, Table A6 in SI). This was followed by *Pollution & Contamination Issues* (i.e., *N*_current_ = 11, *N*_future_ = 7). Interestingly, *Cross-Cutting Issues* was selected as the third biggest issue faced currently (*n* = 10, 17% of participants), although this was the second biggest issue participants expect to face in the next 5 years, mentioned by nearly a quarter of participants (*n* = 14, 24%). Finally, only 5% of participants indicated that *Emerging Societal Issues* were the single biggest issue currently faced by participants (*n* = 3), although two additional participants selected this as the single biggest issue they expect to face in the next 5 years (*n* = 5, 8%) (Fig. 2, Table A6).

Participants provided further rationales for their selection of single biggest issues they currently face and expect to face in the next 5 years. Additional elaborations included rationales related to (i) Land use management, including land use changes from development, (ii) stormwater management, (iii) pollution, (iv) renewable energy, and (v) governance issues including government decision-making and informing the public on environmental issues (see Table A6). For example, one participant noted “In rural counties, people don’t really believe in environmental issues or concerns such as climate change, so this information needs to be presented in a way that does not come off as being “too liberal,” otherwise there will not be buy-in.” In terms of issues they expect to face in the next 5 years, one participant mentioned, for example, “Loss of productive lands to residential and commercial development (including development of land prone to hazards of hurricanes and flooding with little knowledge of hazards provided by land developers).”

### Guidance and assistance

**Fig. 3:**
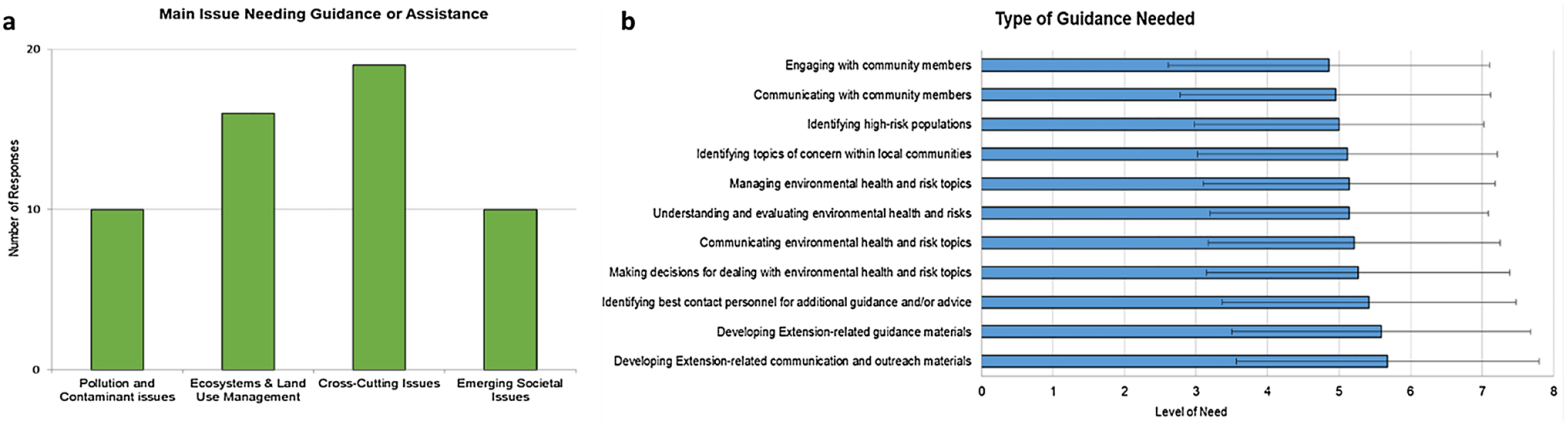
**a** Areas in which participants indicated they needed guidance or assistance to mitigate environmental health and risk issues. Responses to “Of all environmental health and risk categories listed, which one is the biggest issue you need guidance or assistance to mitigate in your community(ies)?” **b** Level of need for guidance or assistance. Responses to “To what degree would you like guidance in the following areas” (1 = Very little, 7 = Very significant amount)

Across all categories of environmental health and risk issues described in the previous sections, participants were asked to identify the single biggest issue they need guidance or assistance. Over one-third of survey respondents indicated a need for guidance or assistance within *Cross-Cutting Issues* (*n* = 19, 35%), followed by *Ecosystems & Land Use Management* (*n* = 16, 29%) (Fig. 3a, Table A7 in SI). There was an equal amount of need expressed for guidance or assistance related to *Pollution and Contamination* and *Emerging Societal Issues* (*n* = 10, 18%, each, respectively). When asked to provide a rationale for their responses, three participants responded with explanations related to land use and concerns regarding development, rural communities dealing with flooding, and PFAS contamination (Table A7).

When study participants were asked to indicate the degree to which they needed guidance, participants indicated that they needed a moderate-to-significant amount of guidance across a range of areas related to assessing, managing, communicating, and making decisions regarding environmental health and risk topics as well as engaging with local communities (Fig. 3b, Table A8 in SI). *Developing Extension-related communication and outreach materials* was the top-ranked need in terms of guidance/assistance (*M* = 5.68, SD = 2.12), closely followed by *Developing Extension-related guidance materials* (*M* = 5.59, SD = 2.09). Next, participants indicated several items to be of moderate importance, including *Identifying best contact personnel for additional guidance and/or advice* (*M* = 5.42, SD = 2.05), *Making decisions for dealing with—*(*M* = 5.27, SD = 2.12), *Communicating—*(*M* = 5.21, SD = 2.04), *Understanding and evaluating*—(*M* = 5.14, SD = 1.95), and *Managing environmental health and risk topics* (*M* = 5.14, SD = 2.04). Other areas that participants indicated they had a moderate amount of need for guidance/assistance included *Identifying topics of concern within local communities* (*M* = 5.12, SD = 2.10), *Identifying high-risk populations* (*M* = 5, SD = 2.03, *Communicating with community members* (*M* = 4.95, SD = 2.17), and *Engaging with community members* (*M* = 4.86, SD = 2.4) (Fig. 3b, Table A8). Participants were also able to indicate if there were additional areas of guidance/assistance that they need that were not listed in the survey. Three participants responded to this open-ended question, and indicated they had a need for guidance/assistance in the following: communicating with news media, identifying the best contact person to forward questions, and to define risk and be aware of Extension materials that are available (Table A8).

**Fig. 4:**
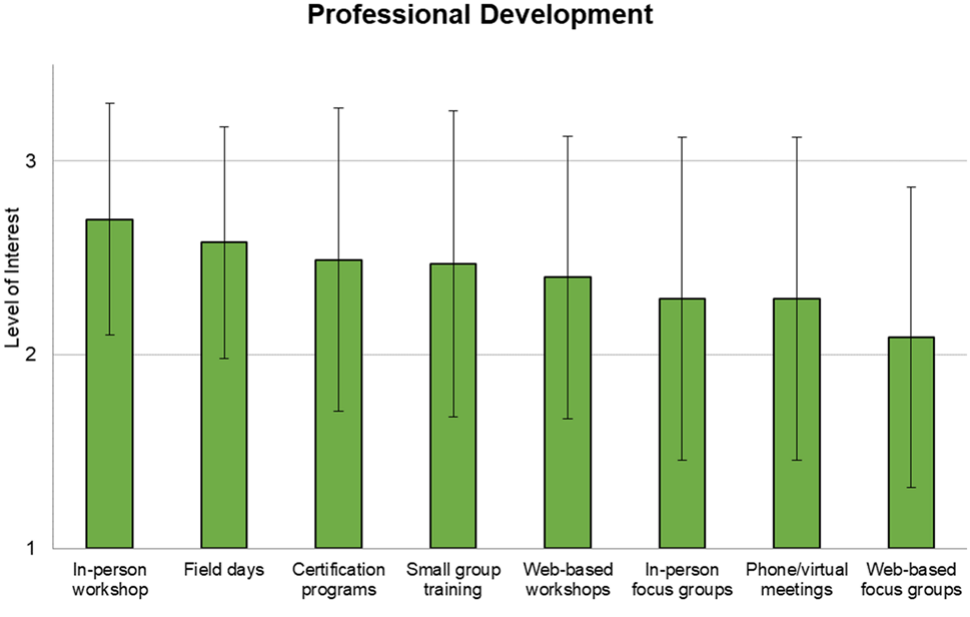
Participant interest in attending professional development events. Responses to “Which of the following professional development events would you be interested in attending in your field in the next year?” (1 = No, 2 = Maybe, 3 = Yes)

Study participants were also asked if they would be interested in attending a variety of professional development events in their field within the next year. Participants largely expressed positive support for attending various professional development events (Fig. 4). On a scale from 1 to 3, where 3 related to a “Yes,” 2 related to “Maybe,” and 1 related to “No” responses, participants were largely in support of attending *In-person workshops* (*M* = 2.7, SD = 0.60), followed by *Field Day events* (*M* = 2.58, SD = 0.60). They were also supportive of attending *Certificate programs* (virtual or in-person) (*M* = 2.49, SD = 0.78), *One-on-one or small group training* (virtual or in-person) (*M* = 2.47, SD = 0.79), and *Web-based focus groups* (*M* = 2.4, SD = 0.73) (Table A9 in SI). They were also somewhat interested in *In-person focus groups* (*M* = * M* = 2.29, SD = 0.83), *Phone/virtual meetings* (*M* = 2.29, SD = 0.83), and *Web-based focus groups* (*M* = 2.09, SD = 0.78) (Table A9).

### Information, collaboration, and communication

**Fig. 5:**
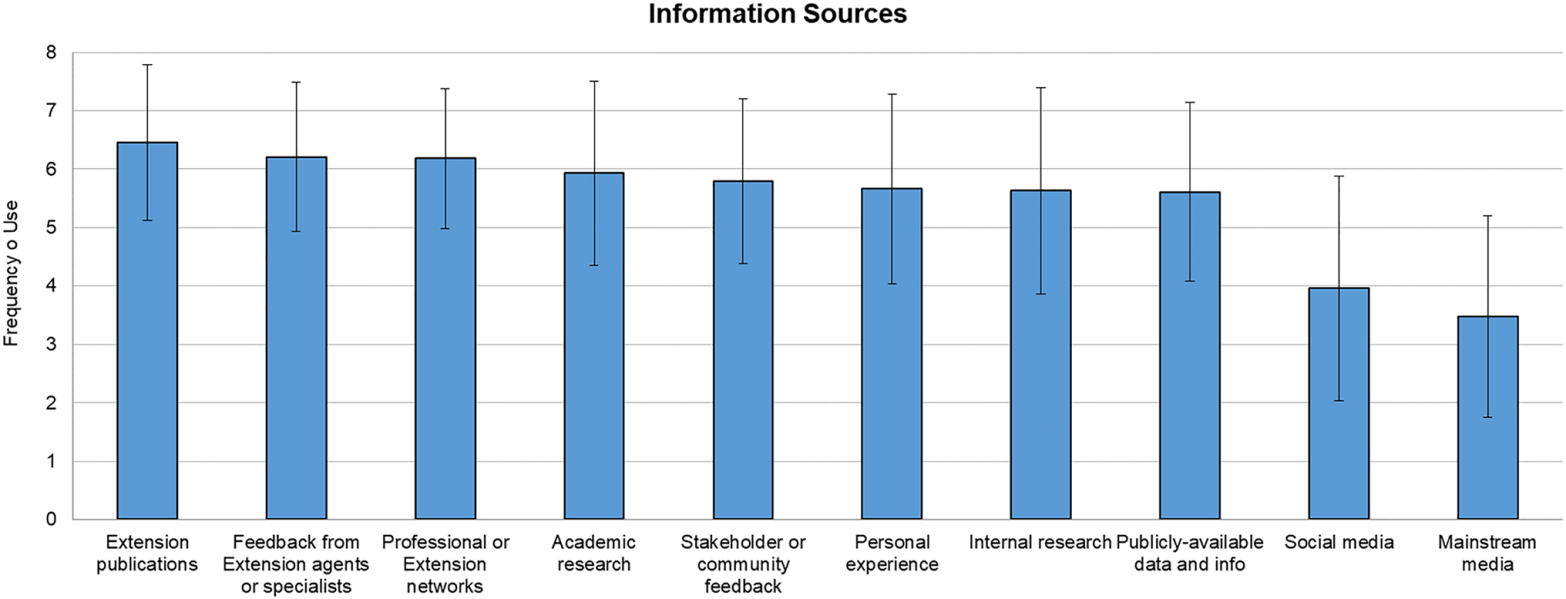
Information sources used by study participants and frequency of use to identify a new or emerging environmental health and risk issue. Responses to “How often do you use each of the following information sources when identifying a new or emerging environmental health and risk issue?” (0 = Do not use; 1 = Rarely use, 7 = Very frequently use)

To identify new or emerging environmental health and risk issues, study participants indicated that they use a range of information sources (Fig. 5, Table A10 in SI). Across all sources, participants indicated that they use *Extension publications* (*M* = 6.45, SD = 1.33), *Feedback from extension agents and specialists* (*M* = 6.21, SD = 1.3), *and Professional or extension networks* (*M* = 6.18, SD = 1.21) regularly to very frequently. Participants indicated that they use *Academic research* (*M* = 5.93, SD = 1.58), *Stakeholder or community feedback* (*M* = 5.79, SD = 1.41), *Personal experienc*e (*M* = 5.66, SD = 1.63), *Internal research* (*M* = 5.63, SD = 1.77), and *Publicly available data and information* (*M* = 5.61, SD = 1.53) often to regularly. Finally, participants indicated they use *Social media* somewhat often (*M* = 3.96, SD = 1.93) and M*ainstream media* sometimes to somewhat often (*M* = 3.48, SD = 1.73). When asked if other information sources were used by participants, no additional responses were provided.

**Fig. 6:**
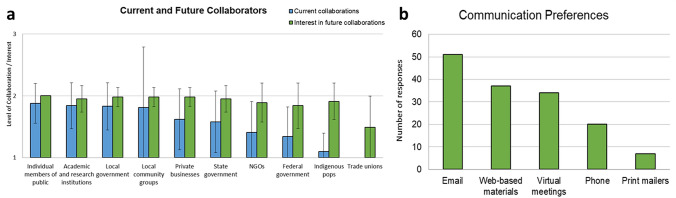
**a** Current and future collaborators identified by study participants. Responses to “Which groups are you currently working with and which groups would you consider working with in the future (either formally or informally) to identify, communicate, or respond to environmental risks?” (1 = No, 2 = Yes). **b** Communication preferences as indicated by study participants. Responses to “How would you prefer to communicate in the future with colleagues?”

In order to identify, communicate with, and/or respond to environmental risks, survey respondents indicated that they currently work with *Individual members of the public* (*M* = 1.88, SD = 0.32), *Academic and research institutions* (*M* = 1.84, SD = 0.37), *Local government* (*M* = 1.83, SD = 0.38), and *Local communities* (*M* = 1.81, SD = 0.98) most often (Fig. 6a, Table A11 in SI). This was followed by some collaborations with *Private businesses* (*M* = 1.62, SD = 0.49), *State government* (*M* = 1.58, SD = 0.5), *NGOs* (*M* = 1.41, SD = 0.5), and *Federal government* (*M* = 1.34, SD = 0.48). Participants indicated that they currently have very minimal collaborations with *Indigenous populations* (*M* = 1.1, SD = 0.3) and no collaborations with *Trade unions* (*M* = 1, SD = 0). One participant also remarked that they currently work with farmers in addition to the groups listed in Fig. 6a. At the same time, survey respondents expressed interest in collaborating with *Individual members of the public* in the future (*M* = 2, SD = 0). Nearly all survey respondents indicated they were interested in collaborating with *Local government* (*M* = 1.98, SD = 0.16), *Local community groups* (*M* = 1.98, SD = 0.16), *Private businesses* (*M* = 1.98, SD = 0.15), *State government* (*M* = 1.95, SD = 0.22), *Academic and research institutions* (*M* = 1.95, SD = 0.22), and *Indigenous populations* (*M* = 1.91, SD = 0.29). Most participants indicated interested in future collaborations with *NGOs* (*M* = 1.91, SD = 0.29) and the *Federal government* (*M* = 1.84, SD = 0.37), while half of survey respondents were interested in collaborating with *Trade unions* (*M* = 1.49, SD = 0.51) in the future (Fig. 6a, Table A11). Finally, participants were asked to indicate their communication preferences. Participants indicated that they most preferred communication through *Email* (*n* = 51), followed by *Web-based materials* (*n* = 37), *Virtual meetings* (*n* = 34), *Phone* (*n* = 20), and then *Print mailers* (*n* = 7) (Fig. 6b, Table A12). No additional communication preferences were indicated by participants.

## Discussion

There were six key themes that emerged in answering our research question, “What are the priority environmental health and risk issues reported by extension agents across North Carolina?” First, the top priority issues according to study participants related to *Water pollution*, *Flooding*, *Natural resources management*, and *Engaging stakeholders*. These issues were not only more highly rated compared to other environmental health and risk issues (between very important to moderately important), but participant responses to the open-comment field questions further emphasized the importance of these areas (e.g., wastewater treatment plants, pollution from animal manure, protection of groundwater supplies, PFAS contamination, connections between water pollution and hurricanes, flooding). Second, building off the previous point, *Natural resources management* was not only one of the top priority areas identified by participants but there were repeated comments throughout the survey on the loss of rural, agricultural, and forest lands to increased residential development and associated impacts on the environment.

These first two themes are similar to and consistent with results from other studies that identify top environmental health and risk priorities in other communities with an emphasis on water quality, natural resource management, and impacts of flooding (often framed within climate change contexts) (Bernhard et al. 2013; Rocks et al. 2017; Wu et al. 2017; Tsui 2020; Wolfson et al. 2020; Fu et al. 2021). Further, these findings are also similar to other studies that emphasize the importance and need to engage stakeholders in approaches to manage environmental health risks (Briggs 2008, O'Brien and Cummins 2008, Wu et al. 2017, Morris et al. 2020). Given the significant population increase in NC within the last decade, and its projected increase in the coming years (North Carolina Office of State Budget and Management 2021), this tension between increased population growth, increased residential development, and loss of agricultural and/or open spaces is expected to continue in the coming years. In fact, study participants indicated that *Ecosystems & Land Use Management* was the single biggest issue they currently face today and expect to face in the future, with supporting comments related to concerns over changing land use patterns due to urban, residential, and commercial development across the state. Participants’ concerns of impacts on ecosystems and land use management also relate to several other studies and reports that have identified environmental degradation and natural resource depletion as one of the top environmental health and risk issues faced at regional and national levels (Tsui 2020; Wolfson et al. 2020; Fu et al. 2021). Concurrently, there is value in noting that some studies report different priorities raised by study respondents from urban and rural communities. In Bernhard et al. (2013), Wu et al. (2017), and Wang et al. (2021), the authors concluded that urban community members focused more on issues of air pollution while rural community members focused more on water and sanitation issues. In our study, we found that more than half of the survey participants identified as having expertise in agriculture and food (likely associated with extension’s heavy agricultural focus groups), which may have influenced the top priority issues identified with more of an emphasis on water quality and natural resource issues.

Third, we find that *Cross-Cutting Issues* that relate to *Engaging stakeholders*, *Assessing*, *Managing*, *Communicating*, and *Assessing risks* were selected as the second biggest issue they expect to face in the next five years and the third single biggest issue participants face currently, mentioned by nearly a quarter of all participants. In fact, there were several comments made throughout the survey that related to challenges of communicating complex risks to diverse stakeholders. Along these lines, other comments related to public trust in scientific information, the politicization of scientific and environmental issues, and other governance matters related to working with community members to manage or mitigate environmental health issues. Unfortunately, these challenges are not entirely surprising, given the intense debates that have often characterized diverse environmental and health risk issues, such as climate change, invasive species management, new technologies used in food/agriculture (e.g., first generation of GMOs), and even the management and governance of pandemics (Kuzma 2018; Kokotovich et al. 2020, Kuzma et al. 2020). In fact, some authors have also suggested that we are in a type of ‘science crisis’ that “undermines the credibility of science and scientists, has multiple origins extending far beyond the domains of public or environmental health research, yet each is affected by it” (Morris et al. 2020). These authors stress the importance of ensuring and maintaining robust connections between environmental health and risk researchers, policy-makers, and civil servants in order to ensure systems-based research to address environmental health challenges. Given the results from our survey, we concur and also emphasize the need for multi-stakeholder collaborations in NC that connect researchers, scientists, policy-makers, extension programs, and community members to address specific environmental health and risk issues in the state.

Fourth, and related to the previous points, participants indicated that they need guidance or assistance in *Cross-Cutting Issues*, followed by *Ecosystems & Land Use Management*. This directly relates to participant responses to previous survey questions, indicating the single biggest issue they currently face and expect to face in the next 5 years. In terms of the type of guidance needed, participants indicated they need a moderate-to-significant amount of guidance across a range of areas related to assessing, managing, communicating, and making decisions regarding environmental health and risk topics as well as engaging with local communities. Participants indicated that they particularly need guidance or assistance in *Developing Extension-related communication and outreach materials*, as well as *Developing Extension-related guidance materials*. This is consistent with participant responses on information sources used to identify new/emerging environmental health and risk issues, whereby extension publications and networks were frequently used sources of information. Other needs for guidance/assistance included *Identifying best contact personnel for additional guidance and/or advice*, *Making decisions for dealing with*, *Communicating*, *Understanding and evaluating*, and *Managing environmental health and risk topics*. These findings indicate that participants need guidance/assistance across a spectrum of needs related to risk governance of environmental health and risk issues, spanning from scientific assessments to community engagement and decision-support. Again, these findings are consistent with the broader risk governance literature that emphasizes the importance of comprehensive efforts to not only understand environmental health and risk issues (through risk assessments and risk analyses), but efforts to manage these risks, communicate risks to diverse stakeholders and actors, and make decisions regarding these risks (e.g., (International Risk Governance Council (IRGC) 2017)).

Our survey also demonstrated, fifth, that while participants need guidance and/or assistance in a range of areas, they are interested in participating in various professional development areas to respond to environmental health and risk issues. Finally, participants indicated that they have active collaborations with several stakeholder groups within academia, government, NGOs, and the public, although they are interested in expanding these collaborations in the future, particularly with *Indigenous populations* and *Trade Unions*. Participants also sought to deepen their engagement with *NGOs*, *Federal government*, *State government*, and *Private businesses*.

Alongside these key themes that emerged, we also recognize that there may be several limitations to our study. First, this study reports on individual views of 66 survey respondents associated with NC State’s extension program. This was a small sample size, and therefore did not allow for statistical tests to be conducted to identify and analyze differences of responses based upon participants’ areas of expertise or regions of the state. In addition, this study did not aim to include other participants from outside of NC State’s extension program, such as environmental non-governmental organizations (NGOs), consumer advocacy groups, regulators or policy-makers, etc., given our focus was on understanding priority needs through extension agents across the state. While we recognize that future studies may improve their sampling to achieve a greater statistical power, we feel that the study design was effective and serves as a vital first-tiered approach that can be expanded to include other stakeholders in subsequent work. Second, we primarily were interested in understanding survey participants’ priorities and needs in terms of environmental health and risk issues, and did not seek to conduct an in-depth risk perception study across respondents’ views of multiple hazards and risks. For this reason, we focused the survey questions on views of how important various issues were to respondents and the community(ies) they serve and areas in which they may need guidance in mitigating environmental health and risk issues. These survey questions used a 7-point scale rather than having survey respondents directly rank different issues, in order to establish the degree of difference between topics as well as to diminish the cognitive burdens of rank-ordering an entire set of topics (i.e., direct ranking can often be mentally tasking and burdensome for respondents, and may present challenges when ranking different topics)(Cummings, 2017b). We also did not aim to provide detailed explanations of the topics and sub-topics included in Table 1 to participants, although we provided brief examples in some sub-topic categories to provide further illustration of environmental health and risk issues in these themes, and included an “other” category to allow participants to indicate a topic/sub-topic not included in Table 1. We further note that the identified priorities and needs that emerged from the study may have been influenced by the areas of expertise of the extension agents who completed the survey, many of which were based in food and agriculture. Third, the survey was disseminated and conducted using an online survey platform, and therefore we recognize that participants needed to have access to an internet connection to access the survey. While this is a potential limitation, especially if emulated in other regions, we assumed that individuals that were professionals working with extension programs would have internet access. Finally, we note that we conducted our study in late April and May 2021 and amid the continuing COVID-19 pandemic, which may have affected some participants’ ability to receive emails and access the survey during this time period.

Overall, results from this work helped to identify top priorities and needs related to a diverse range of environmental health and risk issues in NC, according to the perspectives of the 66 study participants who serve as extension agents. These findings not only can help illustrate top needs, concerns, and priorities of the communities served by the study participants, but they can also help inform future research and outreach strategies to address these priorities. Based on the outcomes of this study, future work may wish to focus on natural resources management and water pollution in the state—two issues that are applicable across the entire state. These issues may be especially important to address now, in light of a growing population and changing land use patterns from increased development in the coming years. In addition, future work may also wish to focus on developing and strengthening multi-stakeholder approaches to identify, manage, and communicate complex risks as they relate to a range of environmental health and risk issues. Researchers, scientists, local government officials, extension agents, and others working with community members may find value in exploring and/or developing new and novel communication mechanisms that rely on increasingly virtual systems, such as web-based stakeholder engagement platforms (e.g., Grieger et al. 2021; Ruzante et al. 2022). Although virtual communication, through web-based platforms, cannot substitute for in-person interactions, they do offer potential benefits of convenience, the ability to reach new or different community members as well as members of the public.

Finally, because this study utilized our existing networks through extension programs that were well established in NC, we find that our approach demonstrates a relatively simple and low-cost mechanism to elicit perspectives and priorities related to environmental health and risk issues that can be leveraged in other states and regions with established stakeholder and community outreach programs. As extension program continue to seek to improve the risk outlooks of their constituencies, they can better inform leadership of the bottom-up risk priorities of stakeholders with local knowledge and experience.

## Conclusions

This study developed and disseminated a written online survey to identify priority issues and needs as they relate to environmental health and risk issues in NC. The survey was distributed to extension agents in NC, and a total of 66 study participants completed the survey. Key outcomes from this work revealed several key themes:

- *Water pollution*, *Flooding*, *Natural resources management*, and *Engaging stakeholders* were top priority issues across all environmental health and risk topics. Continued tensions may be expected across the state when balancing pressures of increased population growth, increased residential development, and loss of agricultural and/or open spaces given the state’s projected increase in population in coming years. These findings are similar to and consistent with results from other studies that identified environmental health and risk priorities, particularly studies with respondents from more rural communities.
- *Cross-Cutting Issues* that relate to *Engaging stakeholders*, *Assessing*, *Managing*, and *Communicating risks* were identified as increasingly important, with several participants noting challenges of communicating complex risks to diverse stakeholders and members of the public. These areas are relevant for a wide range of environmental health and risk issues across the state. These findings continue to support the need for multi-stakeholder collaborations in NC, that connect researchers, scientists, policy-makers, extension programs, and community members to address specific environmental health and risk issues in the state.
- Participants indicated they need a moderate-to-significant amount of guidance across a range of areas related to assessing, managing, communicating, and making decisions regarding environmental health and risk topics as well as engaging with local communities. Such guidance and assistance may be most useful in the form of outreach and extension-based guidance and communication materials. These findings are consistent with the broader risk governance literature, that emphasizes the importance of comprehensive efforts to not only understand environmental health and risk issues through risk assessments and risk analyses, but also efforts to manage, communicate, and make decisions regarding these risks.
- Participants expressed interest in participating in various professional development areas to respond to environmental health and risk issues. Participants indicated that they have active collaborations with several stakeholder groups, although they are interested in expanding these collaborations in the future.

Based on these findings, future work may wish to focus on strengthening natural resources management and mitigating water pollution. Future work may wish to also focus on developing and strengthening multi-stakeholder approaches to identify, manage, and communicate complex risks as they relate to a range of environmental health and risk issues. Overall, we emphasize that adaptive, science-based approaches are needed to respond to the wide range of environmental health and risk challenges in NC and beyond, and these approaches should be coupled with robust community and stakeholder engagement as well as communication efforts. Finally, this study demonstrates a simple, low-cost approach to elicit perspectives and priorities related to environmental health and risks that can be leveraged in other states and regions with established stakeholder and community outreach programs.

## Supplementary Information

Below is the link to the electronic supplementary material.

Supplementary file1 (DOCX 64 kb)

## Footnotes

1 For a complete list of these questions, see Section B of the SI.

2 For a complete list of these questions, see Section C of the SI.

3 For a complete list of these questions, see Section D of the SI.
