# Supplementary material for: Informing environmental health and risk priorities through local outreach and extension
Source: Environ Syst Decis. 2022 Jun 2;42(3):388–401. doi: 10.1007/s10669-022-09864-0 (PMC9161196; doi:10.1007/s10669-022-09864-0)
Supplement: Supplementary file 1 — Supplementary file1 (DOCX 64 kb) [file 10669_2022_9864_MOESM1_ESM.docx]

**Informing Environmental Health and Risk Priorities through Local Outreach and Extension**

Khara Grieger ^a,b,*^, Christopher L. Cummings ^b,c^

^a^ Dept. of Applied Ecology, NC State Extension, North Carolina State University, Campus Box 7617, Raleigh, NC, 27695, USA; ORCID ID 0000-0002-0821-0534; ^*^Corresponding author: [kdgriege@ncsu.edu](mailto:kdgriege@ncsu.edu)

^b^ Genetic Engineering and Society Center, North Carolina State University, Campus Box 7565, Raleigh, NC, 27695, USA;

^c^ Gene Edited Food Program, Iowa State University, 510 Farm House Lane, Ames, Iowa 50011-1054; Contractor, US Army Engineer Research and Development Center

**Supplementary Information (SI)**

**Survey Questions**

The full survey and all questions are included below. The survey was developed and disseminated using Qualtrics, which is an online survey platform.

***Section A. Understanding Priorities***

The state of North Carolina is faced with a variety of environmental health and risk issues, including but not limited to e.g. climate change, PFAS contamination of natural water bodies, and developing sustainable food and agricultural systems in the state. Some issues may be more relevant than others depending upon the county in which you serve.

1. To begin, we would like to know your views of how important each of the following issues is right now to your community(ies) you serve.

|  | **Rating of Importance** | | | | | | |
| --- | --- | --- | --- | --- | --- | --- | --- |
|  | **Not at all important** | **Low importance** | **Slightly important** | **Neutral** | **Moderately important** | **Very important** | **Extremely important** |
| **Soil, Water, Air Pollution & Contaminants** |  |  |  |  |  |  |  |
| Air pollution | 1 | 2 | 3 | 4 | 5 | 6 | 7 |
| Soil pollution | 1 | 2 | 3 | 4 | 5 | 6 | 7 |
| Water pollution (e.g contamination of drinking water and natural waters) | 1 | 2 | 3 | 4 | 5 | 6 | 7 |
| Pollution from industrial chemicals (e.g. PFAS) | 1 | 2 | 3 | 4 | 5 | 6 | 7 |
| Pesticides and pesticide management (e.g. glyphosate) | 1 | 2 | 3 | 4 | 5 | 6 | 7 |
| Pollution from municipal solid waste, (e.g. plastics in the environment) | 1 | 2 | 3 | 4 | 5 | 6 | 7 |
| Other topic related to soil, water, and air pollution (please specify below) | 1 | 2 | 3 | 4 | 5 | 6 | 7 |
| **Ecosystems & Land Use Management** |  |  |  |  |  |  |  |
| Climate change | 1 | 2 | 3 | 4 | 5 | 6 | 7 |
| Flooding, sea level rise, and/or coastal erosion | 1 | 2 | 3 | 4 | 5 | 6 | 7 |
| Biodiversity loss | 1 | 2 | 3 | 4 | 5 | 6 | 7 |
| Deforestation | 1 | 2 | 3 | 4 | 5 | 6 | 7 |
| Fisheries management | 1 | 2 | 3 | 4 | 5 | 6 | 7 |
| Natural resources management | 1 | 2 | 3 | 4 | 5 | 6 | 7 |
| Sustainable agriculture and food security | 1 | 2 | 3 | 4 | 5 | 6 | 7 |
| Other topic related to ecosystems and land use management (please specify below) | 1 | 2 | 3 | 4 | 5 | 6 | 7 |
| **Emerging Issues in Society** |  |  |  |  |  |  |  |
| Micro- and nano-plastics (e.g. in the environment and drinking water sources) | 1 | 2 | 3 | 4 | 5 | 6 | 7 |
| Coal ash and managing coal ash spills | 1 | 2 | 3 | 4 | 5 | 6 | 7 |
| Genetically-modified or genetically-engineered (GE) organisms, (e.g. GE crops or gene drives for conservation) | 1 | 2 | 3 | 4 | 5 | 6 | 7 |
| Nanotechnology and/or nanomaterials (e.g. in food and agriculture products) | 1 | 2 | 3 | 4 | 5 | 6 | 7 |
| Solid waste management (e.g. plastics recycling, composting) | 1 | 2 | 3 | 4 | 5 | 6 | 7 |
| Renewable energy | 1 | 2 | 3 | 4 | 5 | 6 | 7 |
| Other topic related to emerging issues in society (please specify below) | 1 | 2 | 3 | 4 | 5 | 6 | 7 |
| **Cross-Cutting Issues** |  |  |  |  |  |  |  |
| Assessing risks | 1 | 2 | 3 | 4 | 5 | 6 | 7 |
| Managing risks | 1 | 2 | 3 | 4 | 5 | 6 | 7 |
| Communicating risks | 1 | 2 | 3 | 4 | 5 | 6 | 7 |
| Making decisions about risks | 1 | 2 | 3 | 4 | 5 | 6 | 7 |
| Engaging stakeholders | 1 | 2 | 3 | 4 | 5 | 6 | 7 |
| Other cross-cutting issue (please specify below) | 1 | 2 | 3 | 4 | 5 | 6 | 7 |

If there is an additional environmental health and risk-related topic that you think is important and is NOT included in the previous list, please list it here: *_________________________________________________________________*

If you would like to elaborate on any of the environmental health and risk issues that you selected as being highly important, please do so here: *_________________________________________________________________*

1. Of all environmental health and risk issues listed above, which ONE is the biggest issue you feel your community(ies) currently faces?

[RELIST WITH RADIAL BUTTON]

Why?

[OPEN TEXT BOX]

1. Of all environmental health and risk issues listed above, which ONE is the biggest issue you expect to face in the next five years in your community(ies)?

[RELIST WITH RADIAL BUTTON]

Why?

[OPEN TEXT BOX]

***Section B. Guidance and Assistance***

1. Of all environmental health and risk issues listed above, which ONE is the biggest issue you need guidance or assistance to mitigate in your community(ies)?

[RELIST WITH RADIAL BUTTON]

Why?

[OPEN TEXT BOX]

1. To what degree would you like guidance in the following areas?

|  |  | **Rating of desired amount of guidance** | | | | | | |
| --- | --- | --- | --- | --- | --- | --- | --- | --- |
|  | **I do not need any guidance in this area** | **Very**  **little** | **A little** | **Some** | **Fair amount** | **Moderate amount** | **Significant amount** | **Very significant amount** |
| **Developing Extension-related guidance materials** | 0 | 1 | 2 | 3 | 4 | 5 | 6 | 7 |
| **Developing Extension-related communication and outreach materials** | 0 | 1 | 2 | 3 | 4 | 5 | 6 | 7 |
| **Communicating with community members** | 0 | 1 | 2 | 3 | 4 | 5 | 6 | 7 |
| **Engaging with community members** | 0 | 1 | 2 | 3 | 4 | 5 | 6 | 7 |
| **Identifying high-risk populations** | 0 | 1 | 2 | 3 | 4 | 5 | 6 | 7 |
| **Identifying topics of concern within local communities** | 0 | 1 | 2 | 3 | 4 | 5 | 6 | 7 |
| **Identifying best contact personnel for additional guidance and/or advice** | 0 | 1 | 2 | 3 | 4 | 5 | 6 | 7 |
| **Understanding and evaluating environmental health and risks** | 0 | 1 | 2 | 3 | 4 | 5 | 6 | 7 |
| **Managing environmental health and risk topics** | 0 | 1 | 2 | 3 | 4 | 5 | 6 | 7 |
| **Communicating environmental health and risk topics** | 0 | 1 | 2 | 3 | 4 | 5 | 6 | 7 |
| **Making decisions for dealing with environmental health and risk topics** | 0 | 1 | 2 | 3 | 4 | 5 | 6 | 7 |
| **Other (please specify):** | 0 | 1 | 2 | 3 | 4 | 5 | 6 | 7 |

1. Which of the following professional development events would you be interested in attending in your field?

| **Field days** | [Yes/No/Maybe/I don’t know] |
| --- | --- |
| **In-person workshops** | [Yes/No/Maybe/I don’t know] |
| **Web-based workshops** | [Yes/No/Maybe/I don’t know] |
| **In-person focus groups** | [Yes/No/Maybe/I don’t know] |
| **Web-based focus groups** | [Yes/No/Maybe/I don’t know] |
| **Phone/virtual meetings** | [Yes/No/Maybe/I don’t know] |
| **Certification programs (virtual or in-person)** | [Yes/No/Maybe/I don’t know] |
| **One-on-one or small group training (virtual or in-person)** | [Yes/No/Maybe/I don’t know] |
| **Other (please specify):** | [Yes/No/Maybe/I don’t know] |

***Section C. Information and Collaborators***

1. How often do you use each of the following information sources when identifying a new or emerging environmental health and risk issue?

|  |  | **Rating of frequency of use of information sources** | | | | | | |
| --- | --- | --- | --- | --- | --- | --- | --- | --- |
|  | **Do not use** | **Rarely use** | **Somewhat rarely use** | **Sometimes use** | **Somewhat often use** | **Often use** | **Regularly use** | **Very frequent use** |
| **Academic research (e.g. scientific articles)** | 0 | 1 | 2 | 3 | 4 | 5 | 6 | 7 |
| **Internal research (e.g. your own research or Extension-related activities)** | 0 | 1 | 2 | 3 | 4 | 5 | 6 | 7 |
| **Extension publications** | 0 | 1 | 2 | 3 | 4 | 5 | 6 | 7 |
| **Publicly-available data and info** | 0 | 1 | 2 | 3 | 4 | 5 | 6 | 7 |
| **Professional or Extension networks** | 0 | 1 | 2 | 3 | 4 | 5 | 6 | 7 |
| **Stakeholder or community feedback** | 0 | 1 | 2 | 3 | 4 | 5 | 6 | 7 |
| **Feedback from other Extension agents or specialists** | 0 | 1 | 2 | 3 | 4 | 5 | 6 | 7 |
| **Social Media** | 0 | 1 | 2 | 3 | 4 | 5 | 6 | 7 |
| **Mainstream media coverage** | 0 | 1 | 2 | 3 | 4 | 5 | 6 | 7 |
| **Personal experience** | 0 | 1 | 2 | 3 | 4 | 5 | 6 | 7 |
| **Other (please specify)** | 0 | 1 | 2 | 3 | 4 | 5 | 6 | 7 |

1. To identify, communicate, and/or respond to environmental risks, which groups are you currently working with, and which groups would you consider working with in the future, either formally or informally? (Select all that apply)?

| **Local community groups** | [Radial buttons - Currently working with/ consider working with in the future] |
| --- | --- |
| **Individual members of the public** | [Radial buttons - Currently working with/ consider working with in the future] |
| **Local government** | [Radial buttons - Currently working with/ consider working with in the future] |
| **State government** | [Radial buttons - Currently working with/ consider working with in the future] |
| **Federal government** | [Radial buttons - Currently working with/ consider working with in the future] |
| **Trade unions** | [Radial buttons - Currently working with/ consider working with in the future] |
| **Private businesses** | [Radial buttons - Currently working with/ consider working with in the future] |
| **NGOs** | [Radial buttons - Currently working with/ consider working with in the future] |
| **Indigenous populations** | [Radial buttons - Currently working with/ consider working with in the future] |
| **Academic and research institutions** | [Radial buttons - Currently working with/ consider working with in the future] |
| **Other (please specify):** | [Radial buttons - Currently working with/ consider working with in the future] |

***Section D. Respondent Information***

1. What county(ies) do you serve?

[drop down menu]

1. What is your Extension area of expertise?

● Agriculture & Food

● Community

● Forestry Resources

● Health & Nutrition

● Home & Family

● Lawn & Garden

● Soil, Water & Air

● Other (specify)

1. What types of communication mechanisms do you prefer to receive information from colleagues? (check all that apply)

- Email
- Phone
- Virtual meetings
- Print mailers
- Web-based materials
- Other (please specify):

1. Do you have any final comments or thoughts you would like to share with us related to important environmental health & risk topics in the county(ies) you serve? If so, please provide them here: ______________________________________________________________

**Results**

***Section A. Understanding Priorities***

**Table A1.** Importance ratings related to environmental health and risk issues faced by survey participants and communities they serve, within *Soil, Water, Air Pollution, and Contaminant Issues* category. 1 = Not at all important, 2 = Low importance, 3 = Slightly important, 4 = Neutral, 5 = Moderately important, 6 = Very important, 7 = Extremely important. N= number of study participants who filled in a response; Mean = M; Standard deviation = SD.

|  | **Water pollution** | **Pollution from municipal solid waste** | **Soil pollution** | **Pesticides and pesticide management** | **Pollution from industrial chemicals** | **Air pollution** | **Other** |
| --- | --- | --- | --- | --- | --- | --- | --- |
| **N** | 63 | 63 | 63 | 63 | 63 | 63 | 44 |
| **M** | 5.79 | 5.35 | 5.06 | 5 | 4.94 | 4.65 | 4.57 |
| **SD** | 1.54 | 1.52 | 1.51 | 1.81 | 1.92 | 1.72 | 1.54 |

Note: “Other” in Table A1 refers to the following responses from participants:

- Microplastics from biodegradable plastic mulch
- Coal ash
- Stormwater runoff
- Pollution from septic systems in coastal residential development
- Pet waste, CAFOs prone to flooding post hurricane
- Waste from wastewater treatment plants
- I am in a rural community with little to no recycling. Trash litters the sides of the roads. Factories and processing plants put copious amounts of particles in the air nearby
- General litter along roads and streams
- Nonnative species

**Table A2.** Importance ratings related to environmental health and risk issues faced by survey participants and communities they serve, within *Ecosystems & Land Use Management* *Issues* category**.** 1 = Not at all important, 2 = Low importance, 3 = Slightly important, 4 = Neutral, 5 = Moderately important, 6 = Very important, 7 = Extremely important. N= number of study participants who filled in a response; Mean = M; Standard deviation = SD.

|  | **Flood-ing** | **Natural resources manage-ment** | **Sustainable agriculture and food security** | **Climate change** | **Other** | **Deforest-ation** | **Bio-diversity loss** | **Fisheries manage-ment** | **Sea level rise or coastal erosion** |
| --- | --- | --- | --- | --- | --- | --- | --- | --- | --- |
| **N** | 62 | 63 | 63 | 63 | 36 | 62 | 63 | 62 | 62 |
| **M** | 5.6 | 5.56 | 5.38 | 4.9 | 4.61 | 4.53 | 4.49 | 4.34 | 3.82 |
| **SD** | 1.42 | 1.24 | 1.59 | 1.68 | 1.66 | 1.73 | 1.81 | 1.89 | 2.05 |

Note: “Other” in Table A2 refers to the following responses from participants:

- Ecosystem-level habitat protection, including wildlife corridors, etc.
- Development within headwaters of several major river systems leading to the above problems
- Loss of productive land to residential development
- General protection of open space due to expanding urbanization
- Shoreline stabilization
- I live in an area with a lot of timber harvest, so land use is a big factor where education is lacking in my community.
- Weeds and wildlife competing with agriculture crops
- Urban stormwater management
- Litter
- Energy use. The residents here seem to be on a mission to use as much electricity and gasoline as possible. They do not see it as a problem, but I see it as a climate change problem. Also, climate change could be ameliorated by using no-till agriculture, but no one does it here and there is not much financial incentive to try it.
- Equitable food system

**Table A3.** Importance ratings related to environmental health and risk issues faced by survey participants and communities they serve, within *Emerging Societal Issues* category**.** 1 = Not at all important, 2 = Low importance, 3 = Slightly important, 4 = Neutral, 5 = Moderately important, 6 = Very important, 7 = Extremely important. N= number of study participants who filled in a response; Mean = M; Standard deviation = SD.

|  | **Solid waste management** | **Renewable energy** | **Micro- and nano-plastics** | **Genetically engineered organisms** | **Other** | **Coal ash and managing coal ash spills** | **Nanotechnology and/or nanomaterials** |
| --- | --- | --- | --- | --- | --- | --- | --- |
| **N** | 63 | 63 | 63 | 63 | 29 | 63 | 62 |
| **M** | 5.27 | 4.76 | 4.22 | 3.62 | 3.62 | 3.51 | 3.5 |
| **SD** | 1.54 | 1.77 | 2.05 | 1.94 | 1.70 | 1.94 | 1.90 |

Note: “Other” in Table A3 refers to the following responses from participants:

- Cradle-to-grave product lifecycles to minimize solid waste quantity and toxicity
- Carbon sequestration
- Lack of public trust in scientific information

**Table A4.** Importance ratings related to environmental health and risk issues faced by survey participants and communities they serve, within *Cross-Cutting Issues* category**.** 1 = Not at all important, 2 = Low importance, 3 = Slightly important, 4 = Neutral, 5 = Moderately important, 6 = Very important, 7 = Extremely important. N= number of study participants who filled in a response; Mean = M; Standard deviation = SD.

|  | **Engaging stakeholders** | **Managing risks** | **Making decisions about risks** | **Communicating risks** | **Assessing risks** | **Other** |
| --- | --- | --- | --- | --- | --- | --- |
| **N** | 62 | 62 | 62 | 62 | 62 | 27 |
| **M** | 5.53 | 5.19 | 5.13 | 5.08 | 4.92 | 3.93 |
| **SD** | 1.34 | 1.46 | 1.47 | 1.55 | 1.55 | 1.41 |

Note: “Other” in Table A4 refers to the following responses from participants:

- It is important to distinguish between perceived risk and actual risk, the general public doesn't get this. They worry about things they shouldn't and not about things they should worry about.
- If the model for using these cross-cutting issues is similar to the way the Glyphosate concern is being addressed, the concern would be that we do not make that mistake again.
- What are cross cutting issues, what is the definition of risk, what issue requires engagement of stakeholders

**Table A5. Additional responses from participants on environmental health and risk-related topics.** Participants were able to identify a topic of importance otherwise not included in the survey, as well as elaborate on a topic deemed highly important by study participants.

| **Additional topic not on previous list** | **Additional elaboration on highly important topic** |
| --- | --- |
| Farmland preservation | Loss of farm and forest land |
| Managing invasive plants | Some people in my county have not recovered from the last hurricane. Because of climate change, we are expected to get more and longer staying hurricanes |
| Urbanization of rural areas and loss of farm and forest land | Right now PFAS seem to be the biggest concern in part of the county |
| More information regarding the environmental impacts of timber harvest - this is a topic I do not know much about myself but see the practice executed seemingly daily in my community | Protection of groundwater supplies for individual and community wells |
| Wildfire | I noted a high importance regarding environmental impacts on trash/waste. Living in rural NC, there is seemingly little to no emphasis on the benefits of recycling and reducing waste |
| Eco-literacy for all ages | There are 27 identified food deserts in our county (Guilford) -- increasing local food security has been a huge issue for the past 7+ years |

**Table A6. Responses to identify single biggest issue currently facing participants and expect to face in next 5 years.** N= number of study participants who selected a response.

|  | | **Pollution and Contaminant issues** | **Ecosystems & Land Use Management** | **Cross-Cutting Issues** | **Emerging Societal Issues** | **Total** |
| --- | --- | --- | --- | --- | --- | --- |
| **Currently face** | N | 11 | 36 | 10 | 3 | 60 |
|  | % | 18.33 | 60 | 16.67 | 5 | 100% |
| **Expect to face in next 5 years** | N | 7 | 33 | 14 | 5 | 59 |
|  | **%** | 11.86 | 55.93 | 23.73 | 8.47 | 100% |

Note: Further elaboration from participants on responses to single issue they *currently face*:

- Loss of ag and forest land to development
- Over population and urban development
- Streambank management and cleaning major stormwater debris from streams
- Pollution and land use management
- Renewable energy, such as solar
- Government making a lot of decisions
- Unwillingness of citizens to accept scientific information that conflicts with a political interest
- In rural counties, people don’t really believe in environmental issues or concerns such as climate change, so this information needs to be presented in a way that does not come off as being “too liberal,” otherwise there will not be buy-in

Note: Further elaboration from participants on responses to single issue they *expect to face in next five years*:

- Loss of productive lands to residential and commercial development (including development of land prone to hazards of hurricanes and flooding with little knowledge of hazards provided by land developers)
- Over population and urban development
- Changing land uses in our area from rural agricultural to urban development and commercial development
- Renewable energy technology

***Section B. Guidance and Assistance***

**Table A7. Responses to identify single biggest issue participants need guidance or assistance to mitigate.** N= number of study participants who selected a response.

|  | **Pollution and Contaminant issues** | **Ecosystems & Land Use Management** | **Cross-Cutting Issues** | **Emerging Societal Issues** | **Total** |
| --- | --- | --- | --- | --- | --- |
| N | 10 | 16 | 19 | 10 | 55 |
| % | 18.18 | 29.09 | 34.55 | 18.18 | 100% |

Note: Further elaboration from participants on responses to single issue they currently need guidance or assistance to mitigate in their community(ies):

- Residential and urban development at a rapid pace and its influence on the other factors
- Over population and urban development
- Rural communities are struggling with flooding concerns
- PFAS concerns

**Table A8.** Type and level of guidance or assistance needed, as indicated by participants**.** 0=No guidance needed, 1=Very little, 2=A little, 3=Some, 4=Fair amount, 5=Moderate amount, 6=Significant amount, 7=Very significant amount. N= number of study participants who filled in a response; Mean = M; Standard deviation = SD.

| **Type of guidance** | **N** | **M** | **SD** |
| --- | --- | --- | --- |
| Developing Extension-related communication and outreach materials | 56 | 5.68 | 2.12 |
| Developing Extension-related guidance materials | 56 | 5.59 | 2.09 |
| Identifying best contact personnel for additional guidance and/or advice | 56 | 5.42 | 20.5 |
| Making decisions for dealing with environmental health and risk topics | 56 | 5.27 | 2.12 |
| Communicating environmental health and risk topics | 56 | 5.21 | 2.04 |
| Understanding and evaluating environmental health and risks | 56 | 5.14 | 1.95 |
| Managing environmental health and risk topics | 56 | 5.14 | 2.04 |
| Identifying topics of concern within local communities | 57 | 5.12 | 2.10 |
| Identifying high-risk populations | 57 | 5 | 2.03 |
| Communicating with community members | 56 | 4.95 | 2.17 |
| Engaging with community members | 56 | 4.86 | 2.4 |

Note: Further elaboration from participants on responses to guidance they need:

- Communicating with news media
- Person to forward all questions to that has answers
- Define risk and what Extension materials are available

**Table A9.** Participant responses to professional development events they would be interested in attending in their field in the next year**.** 1 = No, 2 = Maybe, 3 = Yes. N= number of study participants who filled in a response; Mean = M; Standard deviation = SD; N_IDK responses = number of study participants who indicated I don’t know for whether they were interested in attending each professional development event type.

|  | **In-person workshop** | **Field days** | **Certification programs (virtual or in-person)** | **One-on-one or Small group training (virtual or in-person)** | **Web-based workshops** | **In-person focus groups** | **Phone / virtual meetings** | **Web-based focus groups** |
| --- | --- | --- | --- | --- | --- | --- | --- | --- |
| **N** | 57 | 55 | 57 | 55 | 57 | 55 | 55 | 55 |
| **M** | 2.7 | 2.58 | 2.49 | 2.47 | 2.4 | 2.29 | 2.29 | 2.09 |
| **SD** | 0.60 | 0.60 | 0.78 | 0.79 | 0.73 | 0.83 | 0.83 | 0.78 |
|  |  |  |  |  |  |  |  |  |
| **N_IDK responses** | 2 | 1 | 3 | 3 | 2 | 3 | 2 | 1 |

Note: In addition to responses of *No, Maybe, and Yes* responses that were scored on a scale from 1-3, participants were also given the option to select *I don’t know* next to whether they were interested in attending each of the professional development event types. As explained in the Methods section, these responses were removed from the scoring scale when calculating mean and standard deviation values, in order to avoid skewing the results. However, the number of participants who indicated *I don’t know* for each professional development event are included in **Table A9** above (see N-IDK values).

***Section C. Information and Collaborators***

**Table A10**. Information sources used by study participants and frequency of use to identify a new or emerging environmental health and risk issue. 0=Do not use, 1=Rarely use, 2=Somewhat rarely use, 3=Sometimes use, 4=Somewhat often use, 5=Often use, 6=Regularly use, 7=Very frequent use. N= number of study participants who filled in a response; Mean = M; Standard deviation = SD.

| **Information sources** | **N** | **M** | **SD** |
| --- | --- | --- | --- |
| Extension publications | 56 | 6.45 | 1.33 |
| Feedback from Extension agents or specialist | 56 | 6.21 | 1.28 |
| Professional or Extension networks | 57 | 6.18 | 1.21 |
| Academic research | 56 | 5.93 | 1.58 |
| Stakeholder or community feedback | 57 | 5.79 | 1.41 |
| Personal experience | 56 | 5.66 | 1.63 |
| Internal research | 56 | 5.63 | 1.77 |
| Publicly-available data and info | 56 | 5.61 | 1.53 |
| Social media | 56 | 3.96 | 1.93 |
| Mainstream media | 56 | 3.48 | 1.73 |

Note: No other information sources were listed by study participants when asked to include other information sources they use to identify a new/emerging environmental health and risk issue.

**Table A11.** Collaborators study participants are currently working with and consider working with in the future to identify, communicate, or respond to environmental risks. 1=No; 2=Yes. N= number of study participants who filled in a response; Mean = M; Standard deviation = SD.

|  | **Current collaborations** | | | **Future collaborations** | | |
| --- | --- | --- | --- | --- | --- | --- |
| **Stakeholder or community groups** | **N** | **M** | **SD** | **N** | **M** | **SD** |
| Individual members of the public | 53 | 1.88 | 0.32 | 41 | 2 | 0 |
| Academic and research institutions | 49 | 1.84 | 0.37 | 42 | 1.95 | 0.22 |
| Local government | 52 | 1.83 | 0.38 | 41 | 1.98 | 0.16 |
| Local community groups | 53 | 1.81 | 0.98 | 41 | 1.98 | 0.16 |
| Private businesses | 47 | 1.62 | 0.49 | 44 | 1.98 | 0.15 |
| State government | 43 | 1.58 | 0.50 | 42 | 1.95 | 0.22 |
| NGOs | 41 | 1.41 | 0.50 | 37 | 1.89 | 0.32 |
| Federal government | 41 | 1.34 | 0.48 | 45 | 1.84 | 0.37 |
| Indigenous populations | 42 | 1.1 | 0.30 | 43 | 1.91 | 0.29 |
| Trade unions | 42 | 1 | 0.00 | 45 | 1.49 | 0.51 |

Note: One participant indicated that they also currently collaborated with *Farmers* in addition to those listed in Table A11. No additional responses were received when participants were asked if there were other collaborators they would be interested in working with in the future.

**Table A12**. Participant responses to communication mechanisms they preferred to use to communicate with colleagues. N= number of study participants who filled in a response; Mean = M; Standard deviation = SD.

|  | **Email** | **Web-based materials** | **Virtual meetings** | **Phone** | **Print mailers** |
| --- | --- | --- | --- | --- | --- |
| **N** | 51 | 37 | 34 | 20 | 7 |
| **% total responses** | 34.2% | 24.8% | 22.8% | 13.4% | 4.7% |

***Section D. Respondent Information***

**Table A13. North Carolina counties served by survey participants.** N= number of study participants who filled in a response.
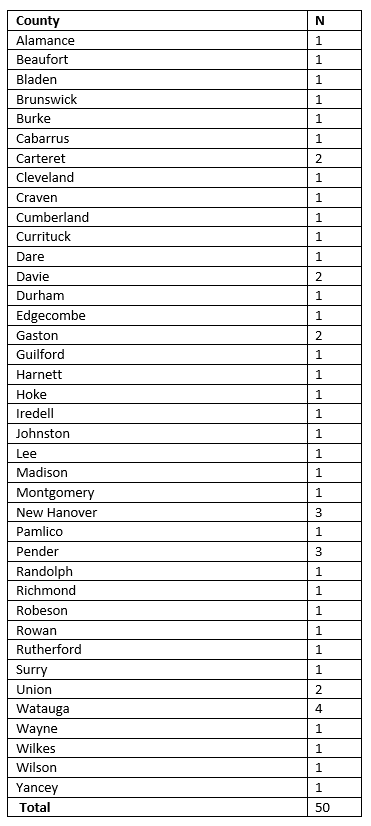


**Table A14.** Areas of expertise as reported by survey participants. N=number of study participants who filled in a response.

|  | **Agriculture & Food** | **Lawn & Garden** | **Community** | **Other** | **Forestry Resources** | **Health & Nutrition** | **Soil, Water, & Air** | **Home & Family** | **Total** |
| --- | --- | --- | --- | --- | --- | --- | --- | --- | --- |
| **N** | 27 | 7 | 6 | 4 | 2 | 2 | 2 | 1 | 51 |
| **%** | 53% | 14% | 12% | 8% | 4% | 4% | 4% | 2% | 100% |

Note: “Other” in Table A13 refers to the following responses from participants:

- Ecosystem protection, Watersheds & Water quality protection
- Natural Resources and Conservation
- Environmental Assessment
- Field Crops
- Commercial and Consumer Horticulture
- Therapeutic Horticulture
- Community & School Gardens, Local Food, Farm to School
- 4-H Youth Development
